# Depression, anxiety, posttraumatic stress disorder and perceived psychosocial care during hospital stay after myocardial infarction: a cross-sectional study

**DOI:** 10.1186/s12872-025-05129-1

**Published:** 2025-09-08

**Authors:** Carolin Swoboda, Paul Gellert, Elisabeth Steinhagen-Thiessen, Ulf Landmesser, Michael A. Rapp, Sandra Düzel, Christian Hering

**Affiliations:** 1 Charité – Universitätsmedizin Berlin, corporate member of Freie Universität Berlin and Humboldt-Universität zu Berlin, Friede Springer – Cardiovascular Prevention Center, Hindenburgdamm 30, 12203 Berlin, Germany; 2 Charité – Universitätsmedizin Berlin, corporate member of Freie Universität Berlin and Humboldt-Universität zu Berlin, Institute of Medical Sociology and Rehabilitation Science, Charitéplatz 1, 10117 Berlin, Germany; 3 Universitätsmedizin Rostock, Institute of Clinical Chemistry and Laboratory Medicine, Ernst-Heydemann-Straße 6, 18057 Rostock, Germany; 4 University of Potsdam, Department of Social and Preventive Medicine, Am Mühlenberg 9, 14476 Potsdam, Germany

**Keywords:** Cardiovascular disease, Heart attack, Psychosocial burden, PTSD, Psychosocial care

## Abstract

**Background:**

Myocardial infarctions (MI) significantly contribute to the global disease burden and are often followed by psychological conditions such as depression, anxiety, and posttraumatic stress disorder (PTSD). These are frequently underrecognized and insufficiently addressed in clinical care. This study aims to investigate the psychosocial impact of MI, identify risk factors for psychological burden following an MI, and gain insight into the perceived psychological care during hospitalization.

**Methods:**

A total of 199 MI patients participated in a cross-sectional online survey conducted between May 15th and August 1st, 2024. Standardized instruments included Depression Anxiety Stress Scale-21 (DASS-21), International Trauma Questionnaire (ITQ), Brief Illness Perception Questionnaire (B-IPQ), ENRICHD Social Support Inventory (ESSI), UCLA 3-Item Loneliness Scale, Brief Resilience Scale (BRS), and Stress and Coping Inventory (SCI). Multiple linear regression models examined associations between psychological burden, psychosocial factors, MI event characteristics, illness perception, history of illness, and perceived psychosocial care during hospitalization.

**Results:**

More than half (58.8%) of MI patients experienced at least one psychological burden, with 37.7% meeting criteria for depression, 46.2% for anxiety, and 18.6% for PTSD. Additionally, 65.9% reported not to be asked about their mental health during hospitalization. Depression was significantly associated with illness perception (β = 0.386), loneliness (β = 0.228), and age (β = − 0.125). Anxiety was associated with illness perception (β = 0.535), multiple MIs (β = 0.168), fear of death (β = 0.117), prior diagnosis of mental disorder (β = 0.113), resuscitation (β = − 0.108), and having no partner (β = − 0.105). PTSD was linked to illness perception (β = 0.371), age (β = − 0.157), fear of death (β = 0.148), multiple MIs (β = 0.122), loneliness (β = 0.149), and social support (β = − 0.139).

**Conclusion:**

The findings emphasize the psychological burden following MI and the need for systematic screening in cardiology to improve patient care.

## Background

Cardiovascular diseases (CVD) and mental disorders are major contributors to the global burden of disease in modern society [1–3]. Projections indicate that by 2030, both CVD and depression will be leading causes of disability worldwide [4–7]. Importantly, cardiovascular and mental health conditions are interlinked with a bidirectional, mutually reinforcing relationship [8–11]. Moreover, patients suffering from both CVD and mental disorders show increased health risks, which can lead to higher morbidity and mortality rates as well as lower quality of life [9, 12–14]. Moreover, the psychological burden significantly impacts recovery, overall well-being, and secondary prevention efforts following MI [14, 18, 21]. Among patients with CVD, the prevalence of mental disorders—particularly depression and posttraumatic stress disorder (PTSD)—is notably high compared to the general population [5, 11, 15–17]. Studies suggest that depression is especially common within the first year after a myocardial infarction (MI), with prevalence estimates ranging from approximately 25–35.4% [18, 19]. Similarly, PTSD is a significant concern with approximately 12% of patients with acute coronary syndrome exhibiting clinically relevant PTSD symptoms [17]. In contrast, the prevalence of anxiety disorders among cardiovascular patients is estimated at 15.5%, a rate comparable to that of the general population [15, 20]. However, research on the association between anxiety and CVD remains less comprehensive, making direct comparisons of prevalence rates across studies challenging [21–25]. The reciprocal relationship between mental and cardiovascular health, and its importance for prevention and care, is recognized by the European Society of Cardiology’s guidelines for the treatment of acute cardiovascular diseases. These guidelines recommend incorporating psychosocial factors into the management of acute cardiovascular conditions [26]. Despite this recognition and growing evidence of the psychological burden following MI, it still seems to be neglected in cardiovascular care. As a result, mental disorders are often inadequately screened, undiagnosed and untreated, in this population [20, 27–32]. Current treatment approaches tend to focus predominantly on the physical manifestations of the disease, frequently overlooking the significant psychological sequelae such as depressive and anxiety symptoms that contribute to overall disease burden [12, 27, 31, 33, 34]. Numerous studies identified associated factors of depression, anxiety, PTSD following MI, e.g., younger age, female gender, and being unpartnered [35–40], as well as having a history of mental disorders [39, 41–45] and multiple MIs in the past [41]. MI-related complications, such as resuscitation due to MI [39, 42, 43, 45], have also been found to be linked to greater psychological distress, by being associated with increased rates of depression, anxiety, PTSD. Additionally, a threatening perception of both heart disease and the MI event itself [43–49], along with psychosocial factors, such as loneliness, lack of social support, low resilience, and inadequate coping mechanisms, contribute to increased rates of depression, anxiety, and PTSD post-MI [35, 39, 50–60]. Despite the growing body of research, little is known about the co-occurrence of depression, anxiety, and PTSD in CVD patients and how various factors are linked to these mental health outcomes. Moreover, it remains unclear to what extent psychosocial screening and mental health care are integrated into routine cardiovascular treatment. While psychosocial distress following MI is well documented, research on actual mental health care utilization and access to psychological support during hospitalization remains scarce. This study aims to investigate the psychosocial burden (depression, anxiety, PTSD) after MI and to examine psychosocial care during hospitalization.

## Methods

### Participants and design

This cross-sectional study was conducted via an online survey between May 15th and August 1st, 2024, in Germany. Eligible participants were adults who had experienced MI, recruited independently of their hospitalization and without restrictions on the time elapsed since the event. Recruitment was carried out through multiple channels, including social media, online forums, medical facilities affiliated with Charité – Universitätsmedizin Berlin, cardiology clinics, through outreach to sports and heart patient groups, and the Deutsche Herzstiftung e.V. (German Heart Foundation), which disseminated study information via its communication channels. The survey was administered using the secure Research Electronic Data Capture (REDCap) platform. The questionnaire was accessed 399 times, with 199 participants completing it in full. Informed consent was obtained online, and the survey was conducted anonymously in accordance with ethical standards and regulations. The study was approved by the Ethics Committee of the Faculty of Medicine at Charité – Universitätsmedizin Berlin (EA2/052/24).

### Measurements

#### Demographics

The online survey collected sociodemographic data, including age, gender (being male, female, divers) and relationship status.

#### Depression, anxiety, and PTSD following MI

Depression and anxiety were assessed using the Depression- and Anxiety-subscales of the Depression Anxiety Stress Scale-21 (DASS-21) [61]. Each subscale consists of 7 items, evaluated on a 4-point Likert scale, measuring the frequency or severity of the participants' experiences over the last week. The final scores – ranging from 0 to 21 – were multiplied by two [62]. Established cutoff values classify depression scores of 9 or lower as ‘normal,’ 10–13 as ‘mild,’ 14–20 as ‘moderate,’ 21–27 as ‘severe,’ and 28 or higher as ‘extremely severe.’ Anxiety scores are categorized as ‘normal’ for values of 7 or lower, ‘mild’ for scores of 8–9, ‘moderate’ for 10–14, ‘severe’ for 15–19, and ‘extremely severe’ for 20 or higher [19]. While these categories provide a broader classification, for descriptive analyses in this study, a cut-off score corresponding to the ‘moderate’ category was applied for both depression and anxiety. Both subscales demonstrated strong internal consistencies in this study (Cronbach *α* = 0.94 in the Depression Subscale, Cronbach *α* = 0.86 in the Anxiety Subscale).

PTSD was assessed using the International Trauma Questionnaire (ITQ) [63]. The ITQ consists of 9 items rated on a 5-point Likert scale (0 = “not at all” to 4 = “very strong”) evaluating traumatic symptoms over the past month. For this study, participants rated all ITQ items specifically in relation to their MI experience, which was defined as the traumatic event. The ITQ measures symptoms of re-experiencing, avoidance, and sense of current threat, along with functional impairment. A PTSD diagnosis requires the endorsement of at least one symptom from each cluster plus one indicator of functional impairment. A dimensional analysis can be performed by summing the core symptom items, excluding those related to functional impairment [63]. The ITQ demonstrated high internal consistency (Cronbach *α* = 0.92) in this study.

#### History of illness

Participants were asked to indicate the timing of their first MI, with responses categorized into predefined time intervals (< 1 year ago, 1–5 years ago, 6–10 years ago, and > 10 years ago). Additionally, they were asked whether they had experienced multiple MIs (“yes” or “no”). If multiple MIs were reported, participants were instructed to focus on the most severe MI in subsequent questions – defined as the MI causing the most emotional distress and life impact. Participants were also queried about any mental disorder history, with the question, “Have you ever been diagnosed with a mental disorder by a qualified professional?” Response options were “Yes, before the (first) heart attack,” “Yes, after the (first) heart attack,” and “No”.

#### MI event characteristics

Two items were developed to assess the event characteristics of the (most impactful) MI. Participants were asked whether resuscitation was performed (“Yes” or “No”) and to rate their fear of death during MI on a 10-point scale (0 = “no fear” to 10 = “extreme fear”).

#### Illness perception

The Brief Illness Perception Questionnaire (B-IPQ) [64] was used to assess illness perception, with modifications to specifically address heart disease. The B-IPQ consists of 8 items rated on a 10-point scale, capturing cognitive and emotional representations of the illness and its perceived threat. The B-IPQ is evaluated by summing the scores of all eight items, resulting in a total score between 0 and 80. For interpretation, the proposed thresholds were applied: <42 points indicate ‘low’, 42–49 points ‘moderate’, and ≥ 50 points ‘high’ threat perception [64, 65]. The B-IPQ demonstrated an acceptable internal consistency (Cronbach *α* = 0.80) in this study.

#### Perceived psychosocial care during hospital stay after MI

To evaluate psychosocial care during hospitalization by hospital staff, four items were developed: (1) “Did the hospital staff ask about your well-being or mental health?”, (2) “Did the hospital staff offer psychological support?”, (3) “Did the hospital staff inform you about the possible psychological effects of a heart attack?”, (4) “Did the hospital staff inform you about counselling services in case of mental distress?”. Response options were “Yes”, “No” and “Not sure/unclear”.

#### Psychosocial factors

Psychosocial factors were assessed using measures of social support, loneliness, resilience, and active coping strategies. Social support was measured using the ENRICHD Social Support Inventory (ESSI) [66], a five-item scale. A total score (5–25) is calculated, with ≤ 18 points or a response of ≤ 3 on at least two items indicating ‘low’ social support [67]. The ESSI demonstrated strong internal consistency (Cronbach *α* = 0.94) in this study. Loneliness was assessed using the UCLA 3-Item Loneliness Scale with scores (range: 3–9) categorized as low (3–5) or moderate to high (6–9) loneliness [68–70]. The scale demonstrated good internal consistency (Cronbach *α* = 0.87) in this study. Resilience was measured using the Brief Resilience Scale (BRS), with six items rated on a five-point Likert scale [71]. Total scores were calculated as the mean (range: 1.00–5.00) and are categorizable into ‘low’ (1.00–2.99), ‘normal’ (3.00–4.30), or ‘high’ resilience (4.31–5.00) based on established cut-offs [72]. The BRS demonstrated a good internal consistency (Cronbach *α* = 0.84) in this study. Active coping strategies were assessed using the Active Coping Strategies subscale of the Stress and Coping Inventory (SCI) [73, 74]. The raw item scores are summed (range: 4–16) and interpreted using the SCI manual’s norm table. Scores of 4–6 indicate ‘low’, 7–10 ‘average’, and 11–16 ‘good’ active stress coping ability. The SCI demonstrated an acceptable internal consistency (Cronbach *α* = 0.76) in this study.

### Statistical analysis

Descriptive statistics were computed for participant characteristics, including frequencies and percentages for categorical variables, and means and standard deviations for continuous variables. Normal distribution of the data was assessed using the Shapiro-Wilk test. As a result, relationships between anxiety, depression, PTSD, and categorical variables were examined using the Mann-Whitney U test and the Kruskal-Wallis test, as appropriate. Multiple regression analyses were conducted to identify factors associated with anxiety, depression, and PTSD following MI. Separate multiple linear regression models were fitted for each outcome (i.e. depression, anxiety, PTSD). Initial examination of our regression models revealed violations of the linearity assumption across all three models. To meet the linearity assumption, dependent variables were log10-transformed after adding 1 to account for a minimum value of zero in the variables (log10(variable + 1)). This transformation reduced skewness and improved linearity, enabling valid regression modeling. Consequently, interpretations shifted from absolute differences to proportional changes in the outcomes, aligning with multiplicative rather than additive effects. Post-transformation, the linearity assumption was satisfied. Subsequently, other key regression assumptions, including independence of residuals, absence of multicollinearity, homoscedasticity, and normality of residuals, were evaluated and met. Outliers were assessed using studentized residuals, leverage points, and Cook’s distance, with no significant issues identified. Both unstandardized (B) and standardized (β) coefficients were reported to interpret the regression models. All analyses were performed using IBM SPSS Statistics for Windows, version 28.0 (IBM Corp., Armonk, NY). Statistical significance was set at *p* < .05.

## Results

### Sample characteristics and prevalence of depression, anxiety and PTSD after MI

A total of *N* = 199 took part in the survey; 43.2% were female and 56.8% were male, with a mean (± *SD*) age of 61.64 years (± 10.75). More than half (58.8%, *n* = 117) of the sample reported psychological distress, with 37.7% meeting the cut-off for depression, 46.2% for anxiety, and 18.6% for PTSD. Female participants were disproportionately affected across all categories (see Table 1). Participants with anxiety had the highest rates of being affected by multiple MI (19.6%), and the most pronounced fear of death during MI (*M* = 7.84, *SD* = 2.41). Illness perception was high across all distressed participants, with participants with PTSD showing the highest perceived threat (*M* = 53.30, *SD* = 9.67). Social support was lowest for participants with PTSD (*M* = 16.03, *SD* = 6.02), while loneliness was highest for participants with depression (*M* = 6.04, *SD* = 2.00). Resilience and active stress coping were low among all distressed participants. Overall, psychosocial care during hospital stay after MI was perceived as insufficient. Among all MI patients, 34.2% reported having been asked about their wellbeing/mental health by hospital staff (Depression: 28.0%, Anxiety: 21.7%, PTSD: 24.3%). 15.1% reported being offered psychological help by hospital staff (Depression: 13.3%, Anxiety: 13.0%, PTSD: 16.2%), and only 11.1% reported being informed about the potential psychological effects of MI by hospital staff (Depression: 10.7%, Anxiety: 7.6%, PTSD: 8.1%). Only 7.0% of the participants reported being informed about counselling services in case of mental distress for mental health by hospital staff (Depression: 5.3%, Anxiety: 5.4%, PTSD: 2.7%). In terms of history of illness, 19.6% of all participants reported a diagnosis of a mental disorder prior to MI, while 16.1% were diagnosed after MI. 64.3% reported no diagnosis of a mental disorder.

**Table 1:** Characteristics of participants

	Total (*N* = 199)	Depression ^a^ (*n* = 75)	Anxiety ^b^ (*n* = 92)	PTSD ^c^ (*n* = 37)
	*n (%)*	*n (%)*	*n (%)*	*n (%)*
Sociodemographics				
Sex				
Female	86 (43.2)	43 (57.3)	50 (54.3)	24 (64.9)
Male	113 (56.8)	32 (42.7)	42 (45.7)	13 (35.1)
Age in Years (*M*, *SD*)	61.64 (10.75)	60.17 (10.05)	58.77 (10.52)	58.89 (9.56)
Relationship status				
No partner ^d^	41 (20.6)	20 (26.7)	24 (26.0)	9 (25.0)
Partnership/married	156 (78.3)	54 (72.0)	67 (72.8)	27 (75.0)
No information	2 (1.0)	1 (1.3)	1 (1.1)	1 (2.4)
MI^a^ Event Characteristics				
Fear of death during MI ^e, f^ (*M*, *SD*)	6.10 (3.35)	6.84 (3.51)	7.84 (2.41)	7.62 (3.11)
Resuscitation				
Yes	36 (18.1)	12 (16.0)	14 (15.2)	10 (27.0)
No	163 (81.9)	63 (84.0)	78 (84.8)	27 (73.0)
Illness perception (M, SD) ^g^	42.96 (13.96)	52.41 (10.65)	52.37 (9.54)	53.30 (9.67)
History of Illness				
Timing of first MI ^e^				
< 1 year ago	47 (23.6)	22 (29.3)	25 (27.2)	15 (27.2)
1–5 years ago	66 (33.2)	31 (41.3)	35 (38.1)	13 (35.1)
6–10 years ago	38 (19.1)	8 (10.7)	18 (19.5)	4 (10.8)
> 10 years ago	48 (24.1)	14 (18.7)	14 (15.2)	5 (13.5)
Multiple MI ^e^				
Yes	34 (17.1)	14 (18.7)	18 (19.6)	7 (18.9)
No	165 (82.9)	61 (81.3)	74 (80.4)	30 (81.1)
Diagnosis of mental disorder				
Before MI ^e^	39 (19.6)	23 (30.7)	27 (29.3)	13 (35.1)
After MI ^e^	32 (16.1)	15 (20.0)	20 (21.7)	8 (21.6)
No diagnosis	128 (64.3)	37 (49.3)	45 (48.9)	16 (43.2)
Perceived psychosocial care during hospital stay after MI ^e^				
Staff asked about wellbeing/mental health				
Yes	68 (34.2)	21 (28.0)	20(21.7)	9 (24.3)
No/Unclear	131 (65.9)	54 (72.0)	72 (78.3)	28 (75.7)
Staff offered psychological help				
Yes	30 (15.1)	10 (13.3)	12 (13.0)	6 (16.2)
No/Unclear	169 (84.9)	65 (86.7)	80 (87.0)	31 (83.8)
Staff informed about the possible impact of MI ^e^ on mental health				
Yes	22 (11.1)	8 (10.7)	7 (7.6)	3 (8.1)
No/Unclear	177 (88.9)	67 (89.3)	85 (92.4)	34 (91.9)
Staff informed about counselling services in case of mental distress				
Yes	14 (7.0)	4 (5.3)	5 (5.4)	1 (2.7)
No/Unclear	185 (92.9)	71 (94.7)	87 (94.6)	36 (97.3)
Psychosocial variables				
Social support ^h^ (*M*, *SD*)	19.57 (5.53)	16.84 (6.70)	18.04 (5.62)	16.03 (6.02)
Loneliness ^i^ (*M*, *SD*)	4.81 (1.98)	6.04 (2.00)	5.49 (1.95)	5.62 (1.74)
Resilience ^j^ (*M*, *SD*)	2.99 (0.92)	2.68 (0.84)	2.61 (0.73)	2.78 (0.92)
Active coping strategies ^k^ (*M*, *SD*)	11.08 (2.41)	10.88 (2.50)	11.03 (2.16)	10.87 (2.45)
Depression, Anxiety, PTSD				
Depression				
Score ≥ 14 (*n*, *%)*	75 (37.7)			
(*M*, *SD*)	11.39 (10.45)			
Anxiety				
Score ≥ 10 (*n*, *%)*	92 (46.2)			
(*M*, *SD*)	10.77 (9.47)			
PTSD				
Fulfilling criteria (*n*, *%)*	37 (18.6)			
Depression, Anxiety and/or PTSD	117 (58.8)			

*M* Mean, *SD* Standard deviation

^a^ Depression assessed with depression scale of DASS-21; Score ≥ 14 (moderate depression)

^b^ assessed with anxiety scale of DASS-21; Score ≥ 10 (moderate anxiety)

^c^ assessed with ITQ

^d^ Single, divorced, widowed

^e^ Myocardial infarction

^f^ scale ranging from 0 to 10, higher scores indicating a greater fear of death during MI

^g^ assessed with BIP-Q

^h^ assessed with ESSI

^I^ assessed with UCLA 3-Item Loneliness Scale

^j^ assessed with BRS

^k^ assessed with SCI

### Relationship between depression, anxiety, PTSD after MI and characteristics of participants

The results of the Mann-Whitney U test indicate significant gender differences in depression, anxiety, and PTSD (see Table 2). Female participants reported significantly higher levels of depression (*p* <.001), anxiety (*p* <.001) and PTSD (*p* <.001) compared to male participants. Additionally, unpartnered individuals exhibited significantly (*p* <.05) higher scores in depression compared to those in relationships. Regarding the perceived psychosocial care during hospital stay after MI, the analysis revealed that individuals who were asked about their wellbeing/mental health showed significantly lower levels in depression (*p* <.05), anxiety (*p* <.001) and PTSD (*p* <.001) than those who were not asked. Finally, Individuals with a diagnosis of a mental disorder prior to their MI showed higher levels in depression (*p* <.001), anxiety (*p* <.001) and PTSD (*p* <.001) than those diagnosed after the infarction or those without a psychological diagnosis.

**Table 2:** Relationship between psychosocial burden and characteristics of participants

	Depression		Anxiety		PTSD	
	*Mdn (IQR)*	*M-W p*	*Mdn (IQR)*	*M-W p*	*Mdn (IQR)*	*M-W p*
Sociodemographics						
Sex						
Female	12.0 (17.0)	**< 0.001**	10.0 (15.0)	**< 0.001**	8.0 (10.25)	**< 0.001**
Male	6.0 (14.0)		6.0 (11.5)		3.0 (8.0)	
Relationship status						
No partner	12.0 (23.0)	**< 0.05**	10.0 (19.0)	0.25	8.0 (8.75)	0.06
Partnership/married	8.0 (14.0)		8.0 (14.0)		4.0 (9.5)	
MI^a^ Event Characteristics						
Resuscitation						
Yes	7.0 (17.0)	0.87	6.0 (13.5)	0.45	6.5 (12.0)	0.46
No	10.0 (16.0)		8.0 (14.0)		5.0 (9.0)	
History of Illness						
Multiple MI ^a^						
Yes	11.0 (20.0)	0.55	11.0 (16.0)	0.18	10.0 (12.0)	0.18
No	8.0 (16.0)		8.0 (14.0)		4.0 (9.0)	
Diagnosis of mental disorder						
Before MI ^a^	17.0 (18.5)	**< 0.001**	17.0 (18.5)	**< 0.001**	10.0 (12.0)	**< 0.001**
After MI ^a^ or No diagnosis	8.0 (14.0)		8.0 (12.0)		4.0 (9.0)	
Perceived psychosocial care during hospital stay after MI ^a^						
Staff asked about wellbeing/mental health						
Yes	6.0 (15.5)	**< 0.05**	6.0 (8.0)	**< 0.001**	2.0 (9.25)	**< 0.001**
No/Unclear	10.0 (16.0)		10.0 (16.0)		7.0 (10.0)	
Staff offered psychological help						
Yes	9.0 (16.0)	0.36	7.0 (16.5)	0.82	3.5 (14.25)	> 0.99
No/Unclear	10.0 (16.0)		8.0 (14.0)		6.0 (9.0)	
Staff informed about the possible impact of MI ^a^ on mental health						
Yes	4.0 (14.0)	0.14	4.0 (8.0)	0.06	2.0 (3.0)	0.12
No/Unclear	10.0 (16.0)		8.0 (15.5)		6.0 (10.50)	
Staff informed about counselling services in case of mental distress						
Yes	2.0 (16.0)	0.09	5.0 (10.5)	0.22	2.0 (4.0)	0.08
No/Unclear	10.0 (16.0)		8.0 (16.0)		6.0 (10.50)	

*M-W p* significance of Mann-Whitney U test, *Mdn* Median, *IQR* interquartile range. Significant results are in bold. ^a^*MI* myocardial infarction

### Multivariate associations between characteristics of participants and depression, anxiety and PTSD after MI

To evaluate the relationships between characteristics of participants and the psychological burden after MI, multiple linear regression models were conducted (see Table 3). For depression, the multiple linear regression model explained 51.3% of the variance (*R²* = 0.513), with an adjusted *R²* of 46.9%. The model showed significant associations with depression (*F*[16, 180] = 11.827, *p* <.001). Illness perception had the strongest association (*β* = 0.386, *B* = 0.014, *p* <.001). Loneliness (*β* = 0.228, *B* = 0.059, *p* <.01) and age (*β* = − 0.125, *B* = − 0.006, *p* <.05) were also significantly associated. For anxiety, the regression model accounted for 58.8% of the variance (*R²* = 0.588), with an adjusted R² of 55.2%. The model indicated significant associations with anxiety (*F*[16, 180] = 16.076, *p* <.001). Illness perception emerged as the strongest association (*β* = 0.535, *B* = 0.018, *p* <.001). Fear of death during MI (*β* = 0.117, *B* = 0.024, *p* <.001) and experiencing multiple MIs (*β* = 0.168, *B* = 0.139, *p* <.01) were also significant. Diagnosis of mental disorder before MI and resuscitation were also significantly associated with anxiety (*β* = 0.113, *B* = 0.131, *p* <.05; *β* = − 0.108, *B* = − 0.127, *p* <.05). Being unpartnered was also significantly associated with anxiety after MI (*β* = − 0.105, *B* = − 0.118, *p* <.05). For PTSD, the model explained 58.4% of the variance (*R²* = 0.584), with an adjusted *R²* of 54.7%. Illness perception had the strongest association (*β* = 0.371, *B* = 0.012, *p* <.001). Age (*β* = − 0.157, *B* = − 0.006, *p* <.01), loneliness (*β* = 0.149, *B* = 0.033, *p* <.05), and fear of death during MI (*β* = 0.148, *B* = 0.019, *p* <.01) were also significantly associated with PTSD. Social support (*β* = − 0.139, *B* = − 0.011, *p* <.05) was negatively associated with PTSD, wheras and multiple MI (*β* = 0.122, *B* = 0.140, *p* <.01) was positively associated with PTSD.

**Table 3:** Multiple linear Regression analysis on Depression (D-DASS-21), Anxiety (A-DASS-21) and PTSD (ITQ)

	Depression			Anxiety			PTSD		
	*B*	*CI (B)*	*β*	*B*	*CI (B)*	*β*	*B*	*CI (B)*	*β*
(Intercept)	0.803*	[0.09, 1.52]		0.425	[−0.17, 1.02]		0.586*	[0.02, 1.15]	
Sociodemographics									
Age	− 0.006*	**[−0.01, 0.00]**	− 0.125*	− 0.004	[−0.01, 0.00]	− 0.097	− 0.006**	**[−0.01, − 0.00]**	− 0.157**
Sex (female)	0.018	[−0.10, 0.14]	0.018	− 0.014	[−0.11, 0.08]	− 0.016	− 0.003	[−0.10, 0.09]	− 0.003
Relationship status (no partner)	− 0.081	[−0.22, 0.06]	− 0.065	− 0.118*	[−0.24, − 0.00]	− 0.105*	− 0.054	[−0.16, 0.06]	− 0.050
MI ^a^ Event Characteristics									
Fear of death during MI ^a^	− 0.006	[−0.02, 0.01]	− 0.039	0.024**	**[0.01, 0.04]**	0.177**	0.019**	**[0.01, 0.03]**	0.148**
Resuscitation (Yes)	− 0.093	[−0.24, 0.05]	− 0.071	− 0.127*	**[−0.25, − 0.01]**	− 0.108*	**− 0.031**	**[−0.14, 0.08]**	**− 0.027**
Perceived psychosocial care during hospital stay after MI ^a^									
Staff asked about wellbeing/mental health (Yes)	0.026	[−0.10, 0.15]	0.024	− 0.061	[−0.17, 0.04]	− 0.064	− 0.094	[−0.19, 0.00]	− 0.104
Staff offered psychological help (Yes)	− 0.065	[−0.26, 0.13]	− 0.046	0.035	[−0.12, 0.19]	0.027	0.093	[−0.06, 0.24]	0.078
Staff informed about the possible impact of MI ^a^ on mental health (Yes)	0.137	[−0.10, 0.37]	0.083	0.040	[−0.15, 0.23]	0.027	0.077	[−0.11, 0.26]	0.055
Staff informed about counselling services in case of mental distress (Yes)	− 0.186	[−0.46, 0.09]	− 0.094	0.001	[−0.23, 0.23]	0.001	− 0.165	[−0.38, 0.05]	− 0.098
History of Illness									
Multiple MIs ^a^ (Yes)	0.117	[−0.02, 0.22]	0.087	0.168**	**[0.05, 0.29]**	0.139**	0.140**	**[0.03, 0.26]**	0.122**
Diagnosis of mental disorder before MI ^a^ (Yes)	0.046	[−0.10, 0.19]	0.036	0.131*	**[0.01, 0.25]**	0.113*	0.062	[−0.05, 0.18]	0.057
Illness Perception of heart disease	0.014***	**[0.01, 0.02]**	0.386***	0.018***	**[0.01, 0.02]**	0.535***	0.012***	**[0.01, 0.02]**	0.371***
Psychosocial factors									
Social support	− 0.012	[−0.03, 0.00]	− 0.128	− 0.008	[−0.02, 0.00]	− 0.097	− 0.011*	**[−0.02, − 0.00]**	− 0.139*
Loneliness	0.059**	**[0.02, 0.10]**	0.228**	**− 0.008**	**[−0.04, 0.02]**	**− 0.033**	0.033*	**[0.00, 0.06]**	0.149*
Resilience	− 0.013	[−0.03, 0.00]	− 0.121	− 0.009	[−0.02, 0.00]	− 0.099	− 0.009	[−0.02, 0.00]	− 0.099
Active coping strategies	0.002	[−0.02, 0.03]	0.011	0.016	[−0.00, 0.03]	0.084	0.010	[−0.01, 0.03]	0.057
*R*^*2*^ (adjusted *R*^*2*^)	0.513 (0.469)			0.588 (0.552)			0.584 (0.547)		

Model = “Inclusion” method in SPSS Statistics, B = Non-standard regression coefficient. *CI* *(B)* *95%* confidence interval for B, The CI was rounded to the second decimal place, *SE (B)* standard error of coefficient B, β = standardised coefficient; Depression, Anxiety, PTSD: log10-transformed

^a^*MI* myocardial infarction

*p <.05. **p <.01. ***p <.001. Significant results are also in bold

## Discussion

The present study aimed to investigate the psychological burden of MI, identify associated factors for depression, anxiety, and PTSD, and gain insights into the perceived psychosocial care during hospital stay after MI. The findings revealed that more than half of the participants suffer from depression, anxiety, and/or PTSD. Still, only up to one third of participants received psychosocial care during hospitalization. Multivariate analysis showed that a threatening illness perception was associated with depression, anxiety, and PTSD. Additionally, younger age and loneliness were linked to depression and PTSD, while multiple MIs and fear of death were associated with anxiety and PTSD. Anxiety was further related to a history of mental disorders, no resuscitation, and being with no partner, whereas PTSD was significantly associated with low social support.

The prevalence rates of the psychological burden observed in the present sample were higher than in previous research [5, 11, 15, 17–20], particularly for depression and anxiety. This discrepancy may have occurred due to differences in assessment timing, as prior studies often collected data during hospitalization [e.g. 18], whereas this study recruited participants online with varying MI histories. Factors such as differences in assessment methods, sample characteristics (e.g., age distribution, socioeconomic status, and pre-existing mental health conditions) or the possibility of prolonged psychological distress accumulation, may explain the higher prevalence found. Regarding sociodemographic factors, age was negatively associated with depression and PTSD, suggesting a greater psychological burden in younger individuals. A possible explanation for this finding is that younger individuals are less prepared to cope with serious health events like MI, and may experience heightened existential anxiety related to career, family, or future plans, which could exacerbate psychological distress. Consistent with this notion, younger patients may perceive acute cardiac events as more threatening, which, in turn, may contribute to the development of posttraumatic symptoms [40]. Moreover, being without a partner also emerged as an important factor, significantly associated with anxiety after MI. The absence of a partner may contribute to an increased psychological burden due to reduced emotional and practical support in coping with different (life-threatening) events. This finding aligns with the broader pattern observed in this study, where social factors play a crucial role in psychological burden. While these findings are in line with prior research [35, 37, 39, 50–52], other psychosocial factors such as resilience and active stress coping did not show significant association with the psychological burden compared to prior research [54–60]. Further, a threatening illness perception was significantly associated with psychological burden after MI, while fear of death was significantly linked to anxiety. These associations align with findings widely discussed in the literature, underscoring the crucial role of subjective perceptions of illness severity in shaping psychological outcomes following MI [43–49]. An unexpected finding of the present study was that participants who did not resuscitation exhibit significantly higher anxiety than those who underwent resuscitation, contradicting the findings of prior studies [41]. A possible explanation could be that resuscitated patients received more intensive care and post-event monitoring, providing reassurance and potentially reducing anxiety. Additionally, the extreme nature of resuscitation might have prompted more social support. In contrast, patients who did not undergo resuscitation may have experienced greater uncertainty and received less social support, leading to higher anxiety. This highlights the need for psychological screenings in all MI patients, not just those with severe medical conditions requiring resuscitation. Consistent with previous research [39, 42, 43], a history of mental disorders prior to MI was found to be a significant factor for anxiety after MI. Moreover, despite the high prevalence of psychological burden, most participants lacked an official diagnosis, suggesting that the mental health of cardiac patients might often be overlooked. In addition to these findings, only a small proportion of participants reported having received psychosocial care during hospitalization, which is in line with previous research [12, 20, 26–34]. While about one third of participants stated that staff asked about wellbeing/mental health, further psychosocial support – such as offering psychological help, information on the psychological impact of MI, and information about counselling services in case of psychological distress—were even more infrequent. Finally, the analysis of the relationship between depression, anxiety, PTSD, and the characteristics of the participants revealed a significant difference between individuals who were asked about their mental health during the hospital stay and those who were not. Specifically, those who received such an inquiry reported lower levels of depression, anxiety, and PTSD. However, multivariate analysis found no significant impact of psychosocial care during hospitalization on psychological burden.

### Limitations

Our study has several limitations that should be considered when interpreting the findings. The cross-sectional design provides only a snapshot of participants’ mental health and limits the ability to draw causal conclusions. Associations found (e.g., between illness perception and psychological distress) cannot clarify directionality or causality. Longitudinal studies are needed to disentangle these bidirectional relationships and establish temporal sequences. Recruitment through online forums, social media platforms, and the German Heart Foundation likely resulted in a self-selected sample with elevated psychological distress and higher health literacy than the general myocardial infarction (MI) population. This introduces selection bias and limits external validity, especially regarding patients who are less digitally engaged or suffer cognitive or physical impairments post-MI. Moreover, the sample size may underrepresent certain subgroups. Data collection relied on retrospective self-reporting of MI-related experiences and psychological symptoms, introducing recall bias. The wide variability in time elapsed since MI events (ranging from less than one year to over ten years) creates temporal heterogeneity and potential confounding. Psychological outcomes may also be influenced by intervening life events and evolving coping strategies, complicating interpretation. Additionally, participants with multiple MIs were instructed to focus on the “most severe” event, a subjective choice that adds further variability and impairs comparability across individuals. Standardized time points or prospective designs could mitigate these issues. Psychosocial care was assessed via four self-constructed, non-validated items with limited psychometric evaluation. Response options such as “Not sure/unclear” may conflate uncertainty with negative responses. Although these variables were retained in the analysis, multivariate models showed no significant impact on psychological outcomes. Consequently, conclusions related to psychosocial care have been tempered to reflect these limitations. An unexpected finding was that non-resuscitated patients reported higher anxiety than resuscitated patients, which contradicts prior research. Without qualitative or longitudinal data to explain care or psychological support differences, this result remains speculative and should be interpreted cautiously. Likewise, several psychosocial predictors (e.g., resilience, active coping) were non-significant, contrasting previous literature. This may reflect sample-specific characteristics or measurement limitations and warrants further investigation. PTSD symptoms were assessed using the ICD-11-based International Trauma Questionnaire (ITQ), which differs from the DSM-5 framework commonly employed in cardiology-psychiatry research. Notably, ICD-11 groups hyperarousal symptoms under “sense of current threat,” potentially underrepresenting some DSM-5 hyperarousal criteria relevant to cardiac populations [75]. Future studies should consider integrative assessments using both diagnostic systems to capture the full clinical spectrum of PTSD symptoms.

### Conclusion

In summary, this study highlights the significant psychological burden experienced by individuals following MI, particularly in terms of depression, anxiety, and PTSD. These findings emphasize the need for more comprehensive psychosocial support during hospitalization, addressing not only the psychological aspects of recovery but also the social and demographic factors that contribute to mental health outcomes. The study underscores the importance of integrating mental health care into routine cardiovascular treatment, as recommended by the European Society of Cardiology. Early identification and management of psychosocial distress, including targeted interventions for factors such as loneliness, illness perception, and pre-existing mental health conditions, are essential for improving both psychological and medical recovery. These insights contribute to the growing understanding of the cardiovascular-mental health connection and provide a foundation for developing more holistic and effective care strategies.

## Data Availability

No datasets were generated or analysed during the current study.

## Abbreviations

B-IPQ: Brief Illness Perception Questionnaire
BRS: Brief Resilience Scale
CVD: Cardiovascular Disease
ESSI: ENRICHD Social Support Inventory
ITQ: Interquartile range
M: Mean
M-W: Mann-Whitney U Test
Mdn: Median
MI: Myocardial Infarction
PTSD: Posttraumatic Stress Disorder
REDCap: Research Electronic Data Capture
SCI: Stress and Coping Inventory
SD: Standard Deviation
